# Chemical Characterization and Bioactivity of Commercial Essential Oils and Hydrolates Obtained from Portuguese Forest Logging and Thinning

**DOI:** 10.3390/molecules27113572

**Published:** 2022-06-02

**Authors:** Ana Ruas, Angelica Graça, Joana Marto, Lídia Gonçalves, Ana Oliveira, Alexandra Nogueira da Silva, Madalena Pimentel, Artur Mendes Moura, Ana Teresa Serra, Ana Cristina Figueiredo, Helena M. Ribeiro

**Affiliations:** 1 Departamento de Biologia Vegetal, Faculdade de Ciências da Universidade de Lisboa, C2, Campo Grande, 1749-016 Lisbon, Portugal; anaruas_@hotmail.com; 2 Research Institute for Medicines (iMed.UL), Faculty of Pharmacy, Universidade de Lisboa, 1649-003 Lisbon, Portugal; angelicagraca@campus.ul.pt (A.G.); jmmarto@ff.ulisboa.pt (J.M.); lgoncalves@ff.ulisboa.pt (L.G.); mpimentel@ff.ulisboa.pt (M.P.); hribeiro@campus.ul.pt (H.M.R.); 3 Laboratory of Microbiological Control, ADEIM—Faculty of Pharmacy, University of Lisbon, Av. Forças Armadas, 1649-019 Lisbon, Portugal; anaoliveira@adeim.pt (A.O.); ansilva@ff.ulisboa.pt (A.N.d.S.); 4 Faculdade de Farmácia, Universidade de Lisboa, 1749-016 Lisbon, Portugal; arturmoura@ff.ulisboa.pt; 5 IBET—Instituto de Biologia Experimental e Tecnológica, Apartado 12, 2780-901 Oeiras, Portugal; tserra@ibet.pt; 6 Instituto de Tecnologia Química e Biológica, Universidade Nova de Lisboa, Av. da República, 2780-157 Oeiras, Portugal; 7 Centro de Estudos do Ambiente e do Mar (CESAM Lisboa), Faculdade de Ciências da Universidade de Lisboa, BV, DBV, C2, Campo Grande, 1749-016 Lisbon, Portugal

**Keywords:** essential oils, hydrolates, chemical composition, antioxidant activity, antimicrobial activity, sensory evaluation

## Abstract

Essential oils (EOs) and hydrolates (Hds) are natural sources of biologically active ingredients with broad applications in the cosmetic industry. In this study, nationally produced (mainland Portugal and Azores archipelago) EOs (11) and Hds (7) obtained from forest logging and thinning of *Eucalyptus globulus*, *Pinus pinaster*, *Pinus pinea* and *Cryptomeria japonica*, were chemically evaluated, and their bioactivity and sensorial properties were assessed. EOs and Hd volatiles (HdVs) were analyzed by GC-FID and GC-MS. 1,8-Cineole was dominant in *E. globulus* EOs and HdVs, and α- and β-pinene in *P. pinaster* EOs. Limonene and α-pinene led in *P. pinea* and *C. japonica* EOs, respectively. *P. pinaster* and *C. japonica* HVs were dominated by α-terpineol and terpinen-4-ol, respectively. The antioxidant activity was determined by DPPH, ORAC and ROS. *C. japonica* EO showed the highest antioxidant activity, whereas one of the *E. globulus* EOs showed the lowest. Antimicrobial activity results revealed different levels of efficacy for *Eucalyptus* and *Pinus* EOs while *C. japonica* EO showed no antimicrobial activity against the selected strains. The perception and applicability of emulsions with 0.5% of EOs were evaluated through an in vivo sensory study. *C. japonica* emulsion, which has a fresh and earthy odour, was chosen as the most pleasant fragrance (60%), followed by *P. pinea* emulsion (53%). In summary, some of the studied EOs and Hds showed antioxidant and antimicrobial activities and they are possible candidates to address the consumers demand for more sustainable and responsibly sourced ingredients.

## 1. Introduction

Nowadays, consumers have a growing interest in substances of natural origin, such as essential oils (EOs), which have been widely used for various purposes. There has been a growing interest from different industries such as pharmaceuticals, cosmetics, and food, in using EOs, mainly due to their biological properties, such as antifungal, antibacterial and antioxidant activities [1]. In the European Union (EU), EOs have been mainly used as flavoring agents in the food industry, in perfumes and aftershaves, in the cosmetics industry, and as functional ingredients in the pharmaceutical industry [2]. In the cosmetics industry, EOs and their isolated constituents are widely used, mainly due to their pleasant scents, as well as their preservative and antioxidant properties [3,4,5]. Moreover, essential oils are used in topical formulations owing to other recognized properties, such as anti-inflammatory, antimicrobial, antioxidant, healing, anti-mutagenic, and anti-aging effects, protection against damage caused by UV-B radiation, and potential use as emollients, dyes, humectants, etc. [5].

In the context of a circular economy, there is an emergent concern in obtaining added value from biomass resulting from forest maintenance, namely from forest logging and thinning. The Mediterranean Forest can provide natural resources that can be exploited and constitute an additional sustainable income to local producers [6,7].

A hydrolate (Hd) is an EO isolation procedure co-product that has a very similar, although less intense, odour compared to its corresponding EO. Unlike EOs, they are water-soluble extracts and can be added to formulas with a high-water content. The characteristics of Hds, especially their biological properties, make them widely used in various industries, such as cosmetics and food. These compounds are promising natural raw materials in many different products [8,9], and several types are already commercially used, mainly as cosmetic and food ingredients. Analysis of their chemical composition show that Hds usually contain less than 1 g/L (i.e., 0.10%) of EO water-soluble compounds. Oxygen-containing compounds are usually dominant in the Hds volatiles, and they may reveal some similarity with EO compositions, although several studies reveal differences, particularly in Hds from hydrocarbon rich EOs [8,9,10].

In this work, EOs and Hds from *Eucalyptus globulus* Labill., *Pinus pinaster* Aiton, *Pinus pinea* L. and *Cryptomeria japonica* D. Don., obtained from forest logging and thinning, were evaluated to gain further insight into their potential use, bioactivity, and likely acceptability in the cosmetic industry. The EOs and their Hds were selected based on their economic and forestry importance in Portugal. In addition, *E. globulus* [11,12,13], *P. pinaster* [14,15,16], *P. pinea* [17,18,19,20] and *C. japonica* [21,22,23,24] EOs and some Hds [8] have shown important biological activities.

The EOs and Hds were obtained from local producers, from mainland Portugal and the Azores archipelago. This study aimed at (1) characterizing the chemical composition of the EOs, and hydrolate volatiles (HdVs), (2) determining their antioxidant and antimicrobial capacity, and (3) assessing only the EOs sensory properties and their acceptability in EOs-topical formulations.

## 2. Results and Discussion

### 2.1. Essential Oil Composition

All EOs were fully chemically characterized (detailed relative amounts of all the identified components are listed in Supplementary Tables S1–S4), although Table 1 reports only their main components (≥5%).

*Eucalyptus globulus*. In total, 44 to 49 compounds were identified in *E. globulus* EOs, accounting for 98–99% of the total composition. Oxygen-containing monoterpenes dominated in all *E. globulus* EOs, ranging from 56 to 72% (Table S1). The main component of the *E. globulus* EOs was 1,8-cineole (eucalyptol), ranging from 49 to 65%. α-Pinene (11–22%), limonene (8–18%), and α-terpenyl acetate (traces-5%) were other relevant compounds (Table 1).

The range of the relative amounts of the main components (1,8-cineole and α-pinene) determined in the present study (Table 1) agrees with the values determined in previous studies for *E. globulus* collected in Portugal (63–70% and 13–20%, respectively) [25,26,27,28,29]. Whereas all samples showed an α-pinene content ≥ 10%, only three had a 1,8-cineole percentage ≥ 60% as specified for the quality assessment of *E. globulus* raw EO by ISO 770:2002 [30].

*Pinus pinaster*. Between 63 and 68 components were identified in *P. pinaster*, accounting for 98–99% of the total composition of EOs (Table S2). *P. pinaster* EOs consisted mainly of monoterpene hydrocarbons (70 to 82%). From the three *P. pinaster* EOs, two were dominated by α-pinene (37–45%), and the third showed similar amounts of α-pinene and β-pinene (28% and 29%, respectively) (Table 1). These results agree with previous reports for *P. pinaster* EOs from Portugal, in which α- and β-pinene were the dominant compounds (25–62% and 20–52%, respectively) [31,32,33].

*Pinus pinea*. Fifty components were identified in *Pinus pinea* EO accounting for 99% of the total composition (Table S3). *P. pinea* EO was dominated by monoterpenes (95%), namely limonene (73%) (Table 1). *P. pinea* EO is known for its chemical homogeneity and despite some percentual variations, the obtained data is comparable to that previously reported by Rodrigues et al. [32] for younger needles collected in Portugal.

*Cryptomeria japonica*. Seventy-nine components were identified in *C. japonica* EO, accounting for 97% of the total composition (Table S4). The main components of *C. japonica* EO were monoterpene hydrocarbons (66%) and diterpene hydrocarbons (16%). α-Pinene (26%), sabinene (18%) and phyllocladene (14%) dominated this EO (Table 1), in agreement with previously reported data for *C. japonica* foliage collected in Azores [27,34,35].

### 2.2. Hydrolates Volatiles Composition

As for all EOs, the hydrolate volatiles (HdVs) were fully chemically characterized, and the detailed relative amounts of all the identified components are listed in Tables S5–S8. Table 2 shows the HdV main components only (≥5%).

*Eucalyptus globulus*. In total, 46–58 constituents were identified in *E. globulus* HdVs (Table S5). Similarly to *E. globulus* essential oils, three of the HdVs were dominated by 1,8-cineole (54–80%) (Table 2), while the fourth sample was dominated by *trans*-pinocarveol (37%). The second main component varied according to the sample, two samples showing high percentages of α-terpineol (17% and 25%), one limonene (7%) and the fourth sample myrtenol (12%).

As detailed in a recent review [8], previous reports indicated 1,8-cineole (62–93%) and α-terpineol (3–17%) as main components of *E. globulus* HdVs [36,37].

*Pinus pinaster*. In *P. pinaster* HdVs, 38 or 42 compounds were identified (Table S6). These HdVs were dominated by oxygen-containing monoterpenes (95% in both cases), namely by α-terpineol (38–44%), followed by verbenone (18–29%) (Table 2).

Although there are reports of *P. cembra* and *P. sylvestris* HdVs [8], to the best of our knowledge no previous study addressed *P. pinaster* HdVs. No study was performed with *P. pinea* HdVs as this hydrolate was not available.

*Cryptomeria japonica*. Forty-four components were identified in *C. japonica* HdVs (Table S7). Oxygen-containing monoterpenes (79%), particularly terpinen-4-ol (56%), dominated *C. japonica* HdVs (Table 2).

These results agree with those reported by Nakagawa et al. [38], which showed that terpinen-4-ol was the main compound of *C. japonica* HdVs, from branches with leaves or just from leaves (32 and 37%, respectively).

### 2.3. Antioxidant Activity of Essential Oils and Hydrolates

#### 2.3.1. DPPH and ORAC Assays

The EOs studied generally showed a weak antioxidant activity determined by the 1,1-diphenyl-2-picrylhydrazyl (DPPH) method. Exceptions were CJ_OE_1_M (23.1 mg/L), which demonstrated a considerable antioxidant activity, followed by Pp_OE_1_G (55.2 mg/L). The lowest antioxidant activity was observed for EG_OE_2_B (647.3 mg/mL). In contrast, the antioxidant activities determined using the Oxygen-Radical Absorbance Capacity (ORAC) method were higher, namely for Pp_OE_3_S (565450.6 µmol TE/g), Pp_OE_2_P (355575.7 µmol TE/g) and Cj_OE_1_M (224877.9 µmol TE/g). With this method, the lower antioxidant activities were obtained for Eg_OE_2_B (53669.2 µmol TE/g) and Eg_OE_5_P (86174.9 µmol TE/g) (Table 3).

*C. japonica* EO had the highest antioxidant capacity determined by the DPPH assay, and the third best when using the ORAC assay (Table 3). Ho et al. [39] used the DPPH method to evaluate the antioxidant activity of *C. japonica* EO obtained from different plant parts (leaf, heartwood, sapwood, bark, and twigs). The reported IC50 values were higher than those obtained in this study, with the sapwood EO showing the highest radical scavenging capability, and the leaf EO having the lowest antioxidant capacity.

One of the *P. pinaster* EO samples, namely Pp_OE_3_S, showed the highest antioxidant capacity, determined by the ORAC assay (Table 3). Mediavilla et al. [7] evaluated the antioxidant activity of forest species EOs, including from *P. pinaster* and *P. sylvestris*, using the ORAC method. *P. pinaster* EO had a higher antioxidant activity than that of *P. sylvestris* EO, which showed the lowest ORAC value of all the species studied. In contrast, the ORAC results obtained herein showed higher antioxidant activity than that described by Mediavilla et al. [7].

The DPHH method did not reveal any measurable antioxidant activity for the Hds, while the results obtained with the ORAC method showed lower values of antioxidant activity of the Hds compared to the respective EOs (Table 3).

As shown in Table 3, the Eg_Hd_2_O sample of *E. globulus* Hd had the highest antioxidant capacity determined by the ORAC assay (1129.7 µmol TE/g). This high antioxidant capacity may be due to the presence of low percentage compounds such as terpinen-4-ol, which was previously reported to have antioxidant efficacy against AAPH radicals [40]. The two samples of *E. globulus* EO had the lowest antioxidant capacity, as shown by both assays. The major components found in these *E. globulus* EO samples were 1,8-cineole and α-pinene, which are described in the literature as compounds with a weak antioxidant activity against DPPH and AAPH radicals [41]. In general, *C. japonica* and *P. pinaster* EOs showed higher antioxidant capacity than that determined for *E. globulus* EOs.

As can be observed from the results, the antioxidant efficiencies determined for the EO samples depend on the methods of evaluation. In general, it was observed that the EO’s antioxidant capacity was higher when using the ORAC method, compared to the DPPH method. The different results between the methods are possibly related to the distinct mechanisms used to evaluate the antioxidant activity. The ORAC method is included in the hydrogen atom transfer (HAT) group of methods, in which there is a competition reaction between antioxidant substances and a fluorescence probe, by a radical [42]. On the other hand, the DPPH method belongs to the electron transfer (ET) group in which a single electron transfer reaction occurs with DPPH reacting by itself, both as a radical and as a probe [43]. Another possible justification for the results obtained is that the ORAC method is more sensitive, being able to detect the antioxidant activity of an extract even when this contains only a small amount of polyphenols. The main advantage of this method is that it combines both the inhibition time and the degree of inhibition of radical generation as it leads the oxidation reaction to completion and uses the area under the curve to quantify antioxidant activity [44].

#### 2.3.2. Intracellular ROS Measurement

In the intracellular reactive oxygen species (ROS) measurement, for a concentration of 10% (*v*/*v*), the EO samples showed a lower capacity to reduce the % ROS, compared to the samples of Hds. In fact, some of the EOs, such as Ppi_OE_1_B and Eg_OE_3_O, even potentiated the formation of ROS (Table 3). The decrease in the % ROS by Hd extracts, for the same concentration, was not significantly different from 1 mg/mL ascorbic acid (*p* ≥ 0.05), except for Eg_Hd_4_O, with a significantly lower (*p* ≤ 0.05) capacity of ROS reduction (47 ± 5%) than that of ascorbic acid. The EO and Hd samples at a lower concentration, 1% (*v*/*v*), were not able to reduce $H_{2}O_{2}$-induced ROS formation.

*C. japonica* Hd showed the best antioxidant capacity against peroxyl radicals (Table 3), possibly because of its main chemical compound, terpinen-4-ol, which is an antioxidant, as suggested by Souza et al. [40]. However, this Hd also contains other antioxidant compounds present in lower amounts, such as β-eudesmol [45]. The second-best antioxidant capacity was observed for a *P. pinaster* Hd sample, a result which is possibly due to the presence of terpinen-4-ol and other compounds, such as thymol [46] and perilla alcohol [47].

### 2.4. Antimicrobial Activity of Essential Oils and Hydrolates

The results of antimicrobial activity are presented in Table 4. Only the EO from *P. pinea* was evaluated, since no Hd was obtained.

The antimicrobial activity of the EOs and Hds was tested against Gram-positive and Gram-negative bacteria, a mold, and a yeast. All *E. globulus* EO samples had antimicrobial activity, although to different extents. Concerning Gram-positive bacteria, all *E. globulus* EO samples showed significant antimicrobial activity against *Bacillus subtilis*, with Eg_OE_5_P presenting the lowest MIC (1.95 µg/mL), while for *Staphylococcus aureus*, the MIC observed when this OE was used was 62.5 µg/mL. Concerning Gram-negative bacteria, most EOs in the tested concentration range were not effective against *Pseudomonas aeruginosa*, except Eg_OE_3_O, with a MIC of 31.25 µg/mL. Eg_OE_2_B and Eg_OE_5_P showed the highest activities against *E. coli* and were also the most active against the pathogenic yeast *Candida albicans*. None of the *E. globulus* samples showed activity against *Aspergillus brasiliensis* (Table 4).

The *P. pinaster* EO samples revealed, in general, an antimicrobial activity lower than that of *E. globulus.* The highest activity was observed on *B. subtillis* with Pp_OE_2_P and Pp_OE_3_S, with MIC values of 15.62 µg/mL. In Gram-negative bacteria, and similarly to the *E. globulus* samples, the *P. pinaster* samples were more active against *E. coli*. No activity against *P. aeruginosa* was observed in the tested concentration range, except for Pp_OE_2_P, for which a high MIC value (500 µg/mL) was obtained. The sample Pp_OE_1_G EO did not have any antimicrobial activity against the tested strains. Furthermore, none of the *P. pinaster* EO samples were active against *A. brasiliensis* (Table 4). The results concerning the *P. pinaster* EO samples revealed that only one of the samples, namely, Pp_OE_2_P, had antimicrobial efficacy against all the strains considered. This may be related to a higher percentage of α-pinene, which was previously mentioned as having antimicrobial properties.

*P. pinea* EO had better activity against *B. subtilis* than against *S. aureus* (Gram-positive) and against *E. coli* than against *P. aeruginosa* (Gram-negative). This sample also had no antifungal activity against *A. brasiliensis*, but was effective against *C. albicans*, with a MIC of 15.62 µg/mL (Table 4).

The *C. japonica* OE did not show any antimicrobial efficacy against any of the tested bacterial and fungal strains. Nevertheless, the variations in the antimicrobial efficacy may be related to other factors that can influence or justify these changes, namely, the culture medium used, the evaluation method, the origin of the botanical species, the plant age, the type of material used (dry or fresh), the amount of EO used in the test, and the isolation technique [48].

The Hds evaluated did not have any detectable antimicrobial activity (data not shown). To the best of our knowledge, the available literature on Hds of the species studied regarding their antimicrobial capacity is scarce. However, the absence of antimicrobial capacity by the Hds may be due to the low concentration of the extracts. Šilha et al. [49] compared Hds of four different plant species, non-concentrated and 50× concentrated and observed that the non-concentrated ones did not have antimicrobial capacity. On the other hand, concentrated hydrolates showed antimicrobial efficacy.

These results agree with what has been described in the literature. For example, Cimanga et al. [50] showed that *S. aureus* ATCC 6538 and *P. aeruginosa* ATCC 9027, were the strains most resistant to the *E. globulus* EO samples. Furthermore, the *B. subtilis* ATCC 6633 strain was considered one of the most susceptible. Hmamouchi et al. [18] showed that, in general, *P. pinaster* EO had higher antimicrobial activity than *P. pinea* EO. Finally, Nakagawa et al. [38] reported that most of the analyzed extracts of *C. japonica* did not show any measurable antimicrobial efficacy.

### 2.5. Sensorial Evaluation

#### Questionnaire Results

The sensorial evaluation was only performed for five (5) EOs, selected according to each plant species, except for *P. pinaster*, which presented two samples with very different odours, which were therefore both included in the study. From the six samples of *E. globulus* EOs, the chosen one was that containing the highest percentage of 1,8-cineole (eucalyptol), since this compound confers most of the odour from *Eucalyptus* species. The hydrolates were not selected due to their weak, much less intense odour.

A total of 100 inexperienced participants were questioned, most of which were between 41 and 50 years old (30%), followed by the group of 18 to 30 years olds (22%) (Table 5).

The under 18 class had the lowest number of respondents (6%). In addition, 67% were female and 43% were male. The participants were divided into three categories based on their education level. The most common education level was higher education (66%), while the least common one was primary education (13%). The countryside was the region with the highest predominance of individuals (70). The remaining 30 individuals belonged to a city environment (Table 5).

In this section, questions were asked regarding the odour of the emulsions provided to the participants (Table 5). The first question in this study was: “How do you evaluate the emulsions’ odour?”. Most participants (46, 38 and 42 individuals) rated *C. japonica* Cj_OE_1_M, *P. pinea* Ppi_OE_1_B and *P. pinaster* Pp_OE_2_P EOs emulsions, respectively, as having a perceptible odour. The *E. globulus* EO emulsion was considered as having the most intense odour among the four available, with 48 responses. The second question was: “In case you identified any odour, how would you classify it?”. Most respondents rated the odours as pleasant and fresh for the Cj_OE_1_M and Ppi_OE_1_B EOs emulsions, with 60 and 53 answers, respectively. The *E. globulus* (Eg_OE_1_G) EO emulsion was considered a very unpleasant odour compared to the other emulsions, with just 36 answers. Most respondents considered the Pp_OE_1_G and Pp_OE_2_P emulsion odours unpleasant, with 41% and 44% percentage values, respectively (Table 6).

The third question was: “In your opinion, the odours of the different emulsions belong to the same plant species?”. As shown in Table 6, most participants (41%) answered “yes”, i.e., they considered that all emulsions belong to the same plant species. The fourth question in this Section was: “Order the emulsions, according to your preference, on a scale of 1–5”. Overall, the Cj_OE_1_M and Ppi_OE_1_B EOs emulsions were the participants’ favourites, with 28 and 24% of individuals, respectively, considering them to have a pleasant odour, and 19% considering these as their favourite odour (Table 6). The aim of the fifth question was to understand if one or more emulsions caused any feeling of well-being. For this, a single question was asked: “Select which emulsion(s) cause you a feeling of physical or mental well-being.” Most of the participants selected the Cj_OE_1_M EO emulsion (20%), while a similar percentage (17%) answered the Eg_OE_1_G EO emulsion or none of the emulsions (Table 7). The sixth and final question in Section 1, related to question five, was “Refer what feelings of well-being the emulsions caused you”. Most participants responded “refreshing” and “relaxing” as the sensations of well-being caused by the emulsions mentioned above (23 and 18%, respectively).

The results from this section are summarized in Table 8. First, participants were asked whether they were likely to purchase a particular product for personal use with the odour of the selected emulsions. The question was: “Rate each of the products below, on a scale of 1–5, considering the probability of buying one with the emulsion’s odour”. Regarding the Cj_OE_1_M emulsion, participants considered that they would be more likely to purchase an air freshener and massage cream with its odour (34 and 33%, respectively) and 18% of volunteers said they would buy it. Perfume and candy with the Cj_OE_1_M emulsion odour were considered by the majority to be the products they would never buy (43 and 49%, respectively). For the Ppi_OE_1_B emulsion, participants considered that they would likely buy air freshener and massage cream with its odour (29 and 34%, respectively) while 12 and 15% of volunteers, respectively, said they would buy such products. Again, perfume and candy with Ppi_OE_1_B emulsion odour were the products most participants would never buy (52 and 43%, respectively).

Regarding the remaining emulsions, participants considered that they would not buy any of the products with such odours, and perfume and candy were the products they would never buy. Finally, a question was asked regarding other possible applicability of the emulsions’ odours. The question was: “Do you consider that the emulsions’ odour has other applicability? If yes, mention which ones”. As shown in Table 9, most participants would use the emulsions odours in cleaning products (10%). However, it should be considered that about 58 individuals did not answer the question, possibly because they do not consider that the emulsions’ odours could have other applications.

In general, the sensory analysis suggests that the participants preferred milder odours than more intense ones. There was a preference for fresher odours, with participants preferring *C. japonica* and *P. pinea* EO emulsions, which were classified as having fresh odours. On the other hand, emulsions with more intense odours such as *E. globulus* EO were considered less pleasant. Regarding a feeling of well-being, the *C. japonica* EO emulsion was the preferred one. In addition to having the highest percentage by itself, this emulsion was almost always mentioned with others. This emulsion was also considered as having the most appreciated/pleasant odour, being the favourite one, together with the *P. pinea* EO emulsion. In contrast, *E. globulus* EO emulsion was the least appreciated and therefore the one with a more unpleasant odour compared to the others. Regarding the possible applications of the emulsions, it was noticeable that the *C. japonica* and *P. pinea* EO odours were the only ones that could be used for air freshener and massage cream. These emulsions’ odours were also the most appreciated by most volunteers. Volunteers showed a preference for fresh and citrus scents made up of compounds such as α and β-pinene, and limonene rather than EO emulsions with more intense odours, dominated by 1,8-cineole.

These results are in accordance with the literature. The main constituents of *P. pinaster* EO are α and β-pinene, which have been reported in several studies as having a fresh, woody, and earthy scent [51,52,53]. α-Pinene is the dominant compound in *C. japonica* EO, while *P. pinea* EO has a high limonene content, which had a strong citrus aroma [4]. In addition, the main component of *E. globulus* EO is 1,8-cineole, which is a colourless liquid with an intense camphor-like odour [52,54].

This study characterized and evaluated the odour organoleptic characteristics of perfumed emulsions. Nevertheless, further studies are needed to evaluate the stability and safety of the prepared EO emulsions, to ensure safe products and consumer satisfaction [55].

## 3. Materials and Methods

### 3.1. Essential Oils and Hydrolates

The essential oils (EOs) and hydrolates (Hds) were obtained from local producers from mainland Portugal and the Azores archipelago (Table 10). The eleven EOs from *Eucalyptus globulus*, *Pinus pinaster, Pinus pinea* and *Cryptomeria japonica* and the seven Hds were stored at −20 °C until analysis. The leaves, needles, and foliage of *E. globulus*, *P. pinaster* and *P. pinea*, and *C. japonica*, respectively, were used to obtain EOs and their Hds.

### 3.2. Hydrolate Volatiles Extraction

Volatiles from hydrolates (HdVs) were obtained by liquid–liquid extraction, using in-lab distilled *n*-pentane, in a ratio of 3 volumes of *n*-pentane per volume of hydrolate. Pentane extracts were concentrated at room temperature under reduced pressure on a rotary evaporator Yamato Hitec RE-51 (Tokyo, Japan). Each extract was then collected in a vial and concentrated to a minimum volume (100 µL), at room temperature, under nitrogen flux, using a blow-down evaporator system.

### 3.3. Essential Oil and Hydrolate Volatiles Composition Analysis

The EOs and the HdVs were analysed by gas chromatography-mass spectrometry (GC-MS) for component identification, and by gas chromatography with flame ionization detector (GC-FID) for components quantification.

#### 3.3.1. Gas Chromatography (GC)-Flame Ionization Detection (FID) Analysis

Gas chromatographic analyses were performed using a Perkin Elmer Clarus 400 gas chromatograph (Perkin Elmer, Shelton, CT, USA) equipped with two flame ionization detectors (FIDs), a data handling system and a vaporizing injector port into which two columns of different polarities were installed: a DB-1 fused-silica column (polydimethylsiloxane, 30 m × 0.25 mm i.d., film thickness 0.25 µm; J & W Scientific, Inc., Rancho Cordova, CA, USA) and a DB-17HT fused-silica column [(50% phenyl)-methylpolysiloxane, 30 m × 0.25 mm i.d., film thickness 0.15 µm; J & W Scientific, Inc., Rancho Cordova, CA, USA]. The oven temperature was programmed at 45–175 °C, at 3 °C/min, subsequently at 15 °C/min up to 300 °C, and then held isothermal for 10 min; injector and detector temperatures were 280 °C and 300 °C, respectively; the carrier gas was hydrogen, adjusted to a linear velocity of 30 cm/s. The samples were injected using split sampling technique at a ratio of 1:50. The percentage composition of the volatiles was computed by the normalization method from the GC peak areas, and calculated as mean values of two injections, from each sample, without using the response factors, in accordance with ISO 7609 [56].

#### 3.3.2. Gas Chromatography-Mass Spectrometry (GC-MS)

The GC-MS unit consisted of a Perkin Elmer Clarus 600 gas chromatograph, equipped with DB-1 fused-silica column (30 m × 0.25 mm i.d., film thickness 0.25 µm; J & W Scientific, Inc., Rancho Cordova, CA, USA), and interfaced with a Perkin-Elmer 600 T mass spectrometer (software version 5.4.2.1617, Perkin Elmer, Shelton, CT, USA). Injector and oven temperatures were as above; the transfer line temperature was 280 °C; the ion source temperature was 220 °C; the carrier gas was helium, adjusted to a linear velocity of 30 cm/s; the split ratio was 1:40; the ionization energy was 70 eV; the scan range was 40–300 u; the scan time was 1 s. The identity of the components was assigned by comparison of their retention indices, calculated in accordance with ISO 7609 [56], with C_8_–C_27_ *n*-alkane indices and with a GC-MS spectra from a lab-made library, created with reference essential oils, laboratory-synthesized components, laboratory isolated compounds and commercially available standards.

### 3.4. Determination of Antioxidant Activity

The antioxidant activity was determined by evaluating the 1,1-diphenyl-2-picrylhydrazyl (DPPH) radical scavenging activity and oxygen radical absorbance capacity (ORAC) according to Ribeiro et al. [57] and Freitas et al. [58], respectively.

#### 3.4.1. DPPH Radical Scavenging Activity

To assess the antioxidant activity, an ethanolic solution containing DPPH radicals with a concentration of 1.60 × $10^{-3}$ mol/L was prepared. Several dilutions in absolute ethanol were prepared from each analyzed sample’s stock solution of EOs (10, 20, 30, 40, 50, 60, 70, 80 µL/µL). The same dilutions were repeated for the hydrolates but in phosphate-buffered saline (PBS).

Several dilutions of the EOs were prepared in ethanol, while the same dilutions of Hds were prepared in PBS. The negative control was an ethanolic solution of DPPH, 8 × $10^{-5}$ M. The positive control was 1 × $10^{-3\;}$ M aqueous solution of ascorbic acid.

The absorbance was measured at 517 nm using a fluorescence microplate reader (FLUOstar BMGLabtech, Ortenberg, Germany). The antioxidant activity was calculated as percentage inhibition of DPPH, using Equation (1)

(1) $$\%\;\mathrm{Inhibition}=\left(\frac{A_{DPPH\;-\;A_{S}}}{A_{DPPH}}\right)\;\times \;100$$

where $A_{DPPH}$ is the absorbance of the DPPH solution and $A_{S}$ is the absorbance of the solution when the EO samples were added. Each experiment was performed in triplicate, and results are expressed as mean ± standard deviation (SD). The half-maximal effective concentration ($EC_{50})$, i.e., the required sample concentration to scavenge 50% of the DPPH radicals present in the solution, was calculated using the GraphPad Prism 5.0 software.

#### 3.4.2. Oxygen Radical Absorbance Capacity (ORAC)

The ORAC method followed the protocol described by Freitas et al. [58]. Briefly, a mixture consisting of 5.18 M from 2,2’azobis (2-methylpropionamidine) dihydrochloride (AAPH) and 4 × $10^{-3}$ mM of disodium fluorescein (DF) (both prepared in 75 mM PBS pH: 7.4) was added to each well of a 96-well black plate. A calibration curve was performed with 6-hydroxy-2,5,7,8-tetramethylchroman-2-carboxylic acid (Trolox), in a range of concentrations from 0 µM to 40 µM. The final dilutions of the EOs and Hds samples used to make the readings were obtained after several attempts, until a concentration within the range of values of the Trolox curve was found. The fluorescence of the samples was read in a microplate reader (FLx800 from Biotek^®^ Instruments, Inc., Winnooski, VT, USA), after a 10 min incubation at 37 °C. The fluorescence (excitation 485 nm, emission 527 nm) was determined every minute, for 40 min at 37 °C.

The fluorimeter control software used was Gen5 (version 3.10, Biotek^®^ Instruments, Inc., Winnooski, VT, USA. 2006). The net areas under the curve (AUC) of the standard (Trolox) and samples were calculated. The standard curve was obtained by plotting Trolox concentrations against the average net AUC of each concentration measurements. The results were expressed in micromoles of Trolox equivalent antioxidant capacity per gram of EO/Hd (µmol TEAC/g EO/Hd). All data were presented as mean ± SD of two replicates.

#### 3.4.3. In Vitro Antioxidant Activity

The ability of EOs and Hds samples to reduce the ROS production was determined using a well-characterized probe, 20,70-dichlorofluorescein diacetate ($H_{2}\text{-}\mathrm{DCFDA};$ Life Technologies, Glasgow, UK), as described by Marques et al. [59] and Carriço et al. [60], with some adaptations. Briefly, the human keratinocytes HaCaT cell line (Cell line Service GmbH, Germany) was seeded at 2 × 10^4^ cells per well in 96-well plates with 100 µL of the cell culture medium per well and incubated at 37 °C for 24 h. Cells were pre-incubated for 30 min with 20 µM of $H_{2}\text{-}\mathrm{DCFDA}$, in the dark, at 37 °C. Then the probe solution was removed, and a fresh medium was added containing the different samples to be tested. Then, 1 mg/mL of ascorbic acid was used as positive control and the culture medium was the negative control. Cells were incubated with the different samples at 10% (*v*/*v*) for 1 h at 37 °C prior to the addition of 500 µM $H_{2}O_{2}$.

The DCF levels were determined by fluorescence (excitation 485 nm, emission 520 nm) in a microplate reader (FLUOstar BMGLabtech, Ortenberg, Germany). Data from six replicates were reported as the relative mean of % ROS reduction determined by relative fluorescence units (RFU) of culture medium with $H_{2}O_{2}$ as 100% and the % ROS reduction as in Equation (2):

(2) $$\left[100-\left(\frac{fluorescence\;of\;sample\;exposed\;cells}{fluorescence\;of\;unexposed\;control\;from\;the\;same\;experiement}\right)\right]\times 100$$

The data were expressed as mean ± SD of experiments (*n* = 8). Statistical evaluation of data was performed using a one-way analysis of variance (ANOVA). Tukey–Kramer multiple comparison test (GraphPad PRISM software, version 5.01, La Jolla, CA, USA) was used to compare the difference between the groups, and differences were considered significant for *p* < 0.05.

### 3.5. Evaluation of Antimicrobial Activity

The minimum inhibition concentration (MIC) was determined to evaluate the antimicrobial activity of the EOs and Hds against Gram-positive and Gram-negative bacteria, yeast, and mold.

#### 3.5.1. Microbial Strains

The microbial strains selected for the study were: *Staphylococcus aureus* ATCC 6538, *Bacillus subtilis* ATCC 6633, *Pseudomonas aeruginosa* ATCC 9027, *Escherichia coli* ATCC 8739, *Candida albicans* ATCC 10,231 and *Aspergillus brasiliensis* ATCC 16404, available at ADEIM/Faculty of Pharmacy, University of Lisbon.

#### 3.5.2. Determination of Minimum Inhibitory Concentration by the Microdilution Method

The MIC determinations for each EO and Hd were performed by the broth microdilution method. Microbial suspensions of each strain were prepared in PBS to a final concentration of 1.5 × 10^8^ CFU/mL for bacterial strains and 1.5 × 10^6^ CFU/mL for the yeast and mold. Then, 100 μL Mueller Hinton (MH) for bacteria and Sabourad dextrose broth (SBD) for the yeast and mold were added to each well of a 96-well microplate. Afterwards, 100 μL of the EO and Hd solutions (1 mg/mL) prepared in the appropriate culture media were added to the first well followed by a twofold serial dilution to obtain final concentrations ranging from 500 mg/mL to 0.48 µg/mL. Finally, 10 µL of the bacterial/fungi suspension diluted in the appropriate culture medium were added to each well to obtain a final concentration of 10^5^ CFU/mL. The microplates were incubated at 35–37 °C for 24 h for bacteria or 48 h for yeast, and at 22 °C for 5 days for *Aspergillus brasiliensis*. Bacterial growth and culture medium were used as controls. The minimal inhibitory concentration (MIC) was defined as the lowest concentration where no visible growth was observed, and growth was monitored by measuring OD_600nm_ in a microplate reader (Varioskan™ multimode microplate reader, Thermo Scientific, Massachusetts, USA). All experiments were performed in triplicate.

### 3.6. Sensory Evaluation

A sensory double-blind evaluation of *E. globulus*, *P. pinaster*, *P. pinea* and *C. japonica* EOs in skin care emulsions was performed by a group of inexperienced volunteers (*n* = 100), females and males aged 18 to 60 years old, with a signed informed consent. The academic objective of the research was reported to the participants, maintaining the privacy of the volunteers and the confidentiality of the information collected. The exclusion criteria included persons who had or have an infection with SARS-CoV-2, respiratory health problems or olfactory diseases in which the olfactory part was affected, which could compromise the sensory questionnaire results.

The emulsions were perfumed using five EOs selected from each species under study, except for *P. pinaster* EO, in which two samples from different producers were chosen. To prepare the emulsions, the oily phase (decyl oleate, cetyl alcohol, ceteareth-11, ceteareth-20 and paraffinum liquidum) and aqueous phase (purified water and glycerin) were heated separately to 75 °C. Then the oily phase was added to the water phase and the system was mixed (130 rpm) with constant agitation in a VMI bench mixer until a 30 °C temperature was reached. Finally, the EOs were added and manually mixed. The emulsions were formulated with 0.5% of EO from the selected samples according to the protocol published by Neves et al. [3]. The excipients used in formulations and the percentage composition of the emulsions prepared with the EOs are described in Table 11.

The formulations of the different samples were coded with different colours: *E. globulus*, Eg_OE_1_G, orange; *P. pinaster,* Pp_OE_1_G, blue; *P. pinaster,* Pp_OE_2_P, purple; *P. pinea*, Ppi_OE_1_B, green; and *C. japonica*, Cj_OE_1_M, pink.

To evaluate the acceptability of all formulations, a questionnaire (Table S1) was answered by each volunteer. The questionnaire was divided into two sections: regarding the Emulsions’ Odour, which aimed to classify emulsions according to the olfactory preferences of the selected volunteers, and concerning the Emulsions’ Applicability with the main objective to evaluate the acceptability of the emulsions considering the probability of purchasing different personal care products (perfume; air freshener; massage cream; toothpaste; shampoo; candy) with the odour and colour of the respective emulsion.

The data collected using the sensory questionnaire during the experimental study were subjected to statistical treatment, using the statistical program IBM^®^ SPSS^®^ software (Statistical Package for the Social Sciences version 27 for Windows 10).

## 4. Conclusions

This study focused on the chemical analysis of essential oils and hydrolates from *Eucalyptus globulus* L., *Pinus pinaster* A., *Pinus pinea* L. and *Cryptomeria japonica* D. Don, as well as on assessing their antioxidant and antimicrobial potential and sensorial properties.

Some of the EOs and Hds showed relevant antioxidant activity and antimicrobial activity. Furthermore, the sensory evaluation revealed the odours favoured by the participants and which products could use such odours. Thus, it can be concluded that essential oils could be used as natural antioxidant substances or cosmetic preservatives, for example. Moreover, such products address the demand for sustainable and responsibly sourced odours accepted by consumers, but further testing will be needed to ensure consumer safety and satisfaction.

## Figures and Tables

**Table 1:** Percentage composition of the main components (≥5%) of *Eucalyptus globulus*, *Pinus pinaster, Pinus pinea* and *Cryptomeria japonica* essential oils (EOs). For sample codes *vide* Materials and Methods Section.

EOs Main Components (≥5%)	RI			Samples			
***Eucalyptus globulus***		**Eg_OE_1_G**	**Eg_OE_2_B**	**Eg_OE_3_O**	**Eg_OE_4_E**	**Eg_OE_5_P**	**Eg_OE_6_S**
α-Pinene	930	13.2	13.3	11.0	21.8	14.7	13.8
1,8-Cineole	1005	65.2	63.2	59.5	53.9	58.2	49.4
Limonene	1009	8.2	17.2	13.7	16.6	12.5	18.0
α-Terpenyl acetate	1334	2.2	t	5.4	0.2	0.8	0.9
***Pinus pinaster***		**Pp_OE_1_G**	**Pp_OE_2_P**	**Pp_OE_3_S**			
α-Pinene	930	27.0	44.6	36.5			
β-Pinene	963	28.0	23.0	18.8			
β-Myrcene	975	11.0	5.0	5.9			
δ-3-Carene	1000	6.6	2.1	1.8			
Limonene	1009	4.5	3.9	3.3			
β-Caryophyllene	1414	4.5	5.0	8.7			
Germacrene-D	1474	6.3	1.7	5.6			
***Pinus pinea***		**Ppi_OE_1_B**					
α-Pinene	930	7.6					
Limonene	1009	72.8					
***Cryptomeria japonica***		**Cj_OE_1_M**					
α-Pinene	930	26.1					
Sabinene	958	18.1					
Phyllocladene	2006	13.8					

RI: In-lab calculated retention index of *n*-alkanes on the DB-1 column. t: traces (˂0.05%).

**Table 2:** Percentage composition of the main components (≥5%) of *Eucalyptus globulus*, *Pinus pinaster* and *Cryptomeria japonica* hydrolate volatiles (HdVs). For samples codes *vide* Materials and Methods Section.

HdVs Main Components (≥5%)	RI		Samples		
***Eucalyptus globulus***		**Eg_Hd_1_G**	**Eg_Hd_2_O**	**Eg_Hd_3_E**	**Eg_Hd_4_P**
1,8-Cineole	1005	80.2	55.5	53.5	4.5
Limonene	1009	7.3	14.1	6.7	1.5
*trans*-Pinocarveol	1106	4.9	0.4	8.0	36.6
*cis*-*p*-2-Menthen-1-ol	1114	t	t	t	4.6
Myrtenal	1153		t	t	5.5
α-Terpineol	1159	2.7	17.2	24.7	5.3
Myrtenol	1168		t	t	12.0
*cis*-Carveol	1202	1.1	0.1	2.8	8.6
***Pinus pinaster***		**Pp_Hd_1_G**	**Pp_Hd_2_P**		
1,8-Cineole	1005	5.0	t		
*cis*-*p*-2-Menthen-1-ol	1114	t	14.0		
*neo*-Isopulegol	1116		14.0		
Terpinen-4-ol	1148	7.5			
*p*-Cymen-8-ol	1148	7.5	t		
α-Terpineol	1159	43.8	38.1		
Verbenone	1164	17.9	28.7		
Perilla alcohol	1274	6.6	t		
Thymol	1275	6.6	t		
***Cryptomeria japonica***		**Cj_Hd_1_M**			
1,8-Cineole	1005	6.3			
Terpinen-4-ol	1148	56.2			
α-Terpineol	1159	4.6			
Phyllocladene	2006	4.8			

RI: In-lab calculated retention index of *n*-alkanes on the DB-1 column. t: traces (˂0.05%).

**Table 3:** Antioxidant capacity of the assessed essential oils and hydrolates. For samples codes *vide* Materials and Methods Section.

Essential Oils	DPPH (IC50, mg/mL)	ORAC (µmol TE/g)	Reduction of ROS * (%)
Eg_OE_1_G	197.6 ± 20.4	113245.9 ± 15003.8	40.0 ± 0.9
Eg_OE_2_B	647.3 ± 5.7	53669.2 ± 8659.3	49.3 ± 0.8
Eg_OE_3_O	151.8 ± 0.0	171891.9 ± 25388.4	−15.7 ± 1.5
Eg_OE_4_E	WA	113884.2 ± 14067.0	27.2 ± 0.8
Eg_OE_5_P	WA	86174.9 ± 9813.9	50.0 ± 0.0
Eg_OE_6_S	246.7 ± 24.5	160532.2 ± 16659.3	6.8 ± 1.2
Pp_OE_1_G	55.2 ± 0.9	161208.7 ± 24896.4	34.3 ± 3.7
Pp_OE_2_P	WA	355575.7 ± 30254.3	29.5 ± 0.5
Pp_OE_3_S	WA	565450.6 ± 70377.8	21.7 ± 1.9
Ppi_OE_1_B	195.7 ± 22.9	165063.9 ± 20907.1	−3.3 ± 1.2
Cj_OE_1_M	23.1 ± 0.2	224877.9 ± 25680.9	83.5 ± 2.8
Hydrolates			
Eg_Hd_1_G	WA	84.1 ± 10.0	81.0 ± 2.3
Eg_Hd_2_O	WA	1129.7 ± 100.6	46.8 ± 5.0
Eg_Hd_3_E	WA	454.6 ± 39.7	-
Eg_Hd_4_P	WA	238.5 ± 24.5	79.2 ± 2.0
Pp_Hd_1_G	WA	212.2 ± 16.9	84.8 ± 1.3
Pp_Hd_2_P	WA	295.1 ± 44.4	80.3 ± 1.9
Cj_Hd_1_M	WA	131.1 ± 10.8	92.8 ± 1.3
Ascorbic Acid	0.04 ± 1.1	-	95.3 ± 0.5

TE: Trolox equivalents. * in vitro ROS reduction generated by 500 µM H_2_O_2_ in HaCaT cell line. WA: Without Activity. ROS: Reactive Oxygen Species.

**Table 4:** Minimum inhibitory concentrations (MICs) of *Eucalyptus globulus*, *Pinus pinaster*, *Pinus pinea* and *Cryptomeria japonica* essential oils (EOs) against Gram-positive and Gram-negative bacteria, yeast, and mold. For sample codes *vide* Materials and Methods Section.

	Minimum Inhibitory Concentrations (MICs) (µg/mL)					
EOs Samples	*Staphylococcus aureus* ATCC 6538	*Bacillus* *subtilis* ATCC 6633	*Pseudomonas aeruginosa* ATCC 9027	*Escherichia* *coli* ATCC 8739	*Candida* *albicans* ATCC 10231	*Aspergillus brasiliensis* ATCC 16404
Eg_OE_1_G	125	31.25	500	15.62	7.81	>500
Eg_OE_2_B	125	31.25	500	3.90	3.90	>500
Eg_OE_3_O	62.5	15.62	31.25	15.62	31.25	>500
Eg_OE_4_E	125	15.62	500	62.5	31.25	>500
Eg_OE_5_P	62.5	1.95	500	3.90	3.90	>500
Eg_OE_6_S	62.5	15.62	500	15.62	7.81	>500
Pp_OE_1_G	>500	>500	>500	>500	>500	>500
Pp_OE_2_P	31.25	15.62	500	15.62	62.5	>500
Pp_OE_3_S	>500	15.62	>500	125	125	>500
Ppi_OE_1_B	62.5	7.81	>500	125	15.62	>500
Cj_OE_1_M	>500	>500	>500	>500	>500	>500

**Table 5:** Sociodemographic characteristics of the 100 volunteers.

Sociodemographic Characteristics	All Samples (*n* = 100) *n* (%)
Age range (years)	
<18	6 (6%)
18–30	22 (22%)
31–40	13 (13%)
41–50	30 (30%)
51–60	20 (20%)
>60	9 (9%)
Gender	
Female	67 (67%)
Male	33 (33%)
Education	
Primary education	13 (13%)
Secondary education	26 (26%)
Higher education	61 (61%)
Region	
Countryside	70 (70%)
City	30 (30%)

**Table 6:** Participants responses regarding the characterization of emulsions odour.

Section 1. Emulsions’ Odour. Odoriferous Characterization of Emulsions			N (%)		
Evaluation of odours	Cj_OE_1_M	Ppi_OE_1_B	Eg_OE_1_G	Pp_OE_1_G	Pp_OE_2_P
1. Without odour	3 (3%)	1 (1%)	0 (0%)	15 (15%)	13 (13%)
2. Slightly perceptible	37 (37%)	8 (8%)	4 (4%)	40 (40%)	28 (28%)
3. Perceptible	46 (46%)	38 (38%)	15 (15%)	28 (28%)	42 (42%)
4. Very perceptible	11 (11%)	34 (34%)	33 (33%)	14 (14%)	11 (11%)
5. Intense odour	3 (3%)	19 (19%)	48 (48%)	3 (3%)	6 (6%)
Classification of odours	Cj_OE_1_M	Ppi_OE_1_B	Eg_OE_1_G	Pp_OE_1_G	Pp_OE_2_P
1. Very unpleasant	0 (0%)	9 (9%)	36 (36%)	4 (4%)	5 (5%)
2. Unpleasant	22 (22%)	25 (25%)	27 (27%)	41 (41%)	44 (44%)
3. Pleasant and hot odour	15 (15%)	12 (12%)	15 (15%)	10 (10%)	13 (13%)
4. Pleasant and fresh odour	60 (60%)	53 (53%)	22 (22%)	30 (30%)	27 (27%)
Ranking in order of preference	Cj_OE_1_M	Ppi_OE_1_B	Eg_OE_1_G	Pp_OE_1_G	Pp_OE_2_P
1. Hateful Odour	3 (3%)	8 (8%)	32 (32%)	9 (9%)	11 (11%)
2. Unpleasant Odour	17 (17%)	18 (18%)	23 (23%)	41 (41%)	43 (43%)
3. Neither pleasant nor unpleasant	33 (33%)	31 (31%)	13 (13%)	30 (30%)	22 (22%)
4. Pleasant Odour	28 (28%)	24 (24%)	20 (20%)	18 (18%)	14 (14%)
5. Favourite Odour	19 (19%)	19 (19%)	12 (12%)	2 (2%)	10 (10%)
Do you think that emulsions belong to the same plant species?					
Positive answers					41 (41%)
Uncertainly answers					34 (34%)
Negative answers					25 (25%)

**Table 7:** Participants responses about the feelings of well-being caused by the emulsions’ odour.

Section 1. Emulsions’ Odour. Feelings of Well-Being Caused by the Emulsions’ Odour	N (%)
Emulsions that caused feelings of well-being	
Ppi_OE_1_B and Eg_OE_1_G	2 (2%)
Cj_OE_1_M, Pp_OE_1_G and Pp_OE_2_P	3 (3%)
Cj_OE_1_M	20 (20%)
Cj_OE_1_M and Pp_OE_1_G	1 (1%)
Eg_OE_1_G and Pp_OE_2_P	2 (2%)
Cj_OE_1_M and Pp_OE_2_P	2 (2%)
Cj_OE_1_M and Ppi_OE_1_B	5 (5%)
Pp_OE_2_P	6 (6%)
Ppi_OE_1_B	11 (11%)
Cj_OE_1_M, Ppi_OE_1_B and Eg_OE_1_G	5 (5%)
Eg_OE_1_G	17 (17%)
Cj_OE_1_M, Eg_OE_1_G, Pp_OE_2_P and Pp_OE_1_G	1 (1%)
Cj_OE_1_M and Eg_OE_1_G	1 (1%)
Pp_OE_1_G and Pp_OE_2_P	2 (2%)
Ppi_OE_1_B and Pp_OE_2_P	1 (1%)
Cj_OE_1_M, Ppi_OE_1_B and Pp_OE_1_G	2 (2%)
Cj_OE_1_M, Ppi_OE_1_B, Pp_OE_1_G and Pp_OE_2_P	1 (1%)
Pp_OE_1_G	1 (1%)
None	17 (17%)
Feelings of well-being	
Refreshing	23 (23%)
Decongestant	15 (15%)
Decongestant, Stimulating and Refreshing	2 (2%)
Decongestant and Refreshing	4 (4%)
Relaxing, Decongestant and Refreshing	7 (7%)
Relaxing	18 (18%)
Relaxing and Stimulating	1 (1%)
Stimulating and Refreshing	1 (1%)
Relaxing and Refreshing	4 (4%)
Stimulating	3 (3%)
Relaxing, Stimulating and Refreshing	1 (1%)
Relaxing and Decongestant	2 (2%)
Decongestant and Stimulating	2 (2%)
None	17 (17%)

**Table 8:** Participants responses to purchasing a product with different emulsions’ odours.

Section 2. Applicability’s of Emulsions’ Odour				N (%)		
Purchasing a Product with Emulsions’ Odours	Perfume	Air Freshener	Massage Cream	Toothpaste	Shampoo	Candy
Probability of buying a product with Cj_OE_1_M odour						
1. Would never buy	43 (43%)	12 (12%)	13 (13%)	32 (32%)	20 (20%)	49 (49%)
2. Unlikely	28 (28%)	31 (31%)	25 (25%)	36 (36%)	31 (31%)	37 (37%)
3. Likely	19 (19%)	34 (34%)	33 (33%)	17 (17%)	27 (27%)	8 (8%)
4. Quite likely	4 (4%)	5 (5%)	11 (11%)	4 (4%)	7 (7%)	2 (2%)
5. Would buy	6 (6%)	18 (18%)	18 (18%)	11 (11%)	15 (15%)	4 (4%)
Probability of buying a product with Ppi_OE_1_B odour						
1. Would never buy	52 (52%)	22 (22%)	16 (16%)	26 (26%)	31 (31%)	43 (43%)
2. Unlikely	29 (29%)	26 (26%)	29 (29%)	27 (27%)	25 (25%)	27 (27%)
3. Likely	11 (11%)	29 (29%)	34 (34%)	27 (27%)	23 (23%)	19 (19%)
4. Quite likely	3 (3%)	11 (11%)	6 (6%)	9 (9%)	7 (7%)	4 (4%)
5. Would buy	5 (5%)	12 (12%)	15 (15%)	11 (11%)	14 (14%)	7 (7%)
Probability of buying a product with Eg_OE_1_G odour						
1. Would never buy	63 (63%)	36 (36%)	38 (38%)	44 (44%)	37 (37%)	53 (53%)
2. Unlikely	20 (20%)	22 (22%)	25 (25%)	30 (30%)	28 (28%)	20 (20%)
3. Likely	9 (9%)	20 (20%)	17 (17%)	13 (13%)	17 (17%)	8 (8%)
4. Quite likely	4 (4%)	5 (5%)	7 (7%)	7 (7%)	7 (7%)	5 (5%)
5. Would buy	4 (4%)	17 (17%)	13 (13%)	6 (6%)	11 (11%)	14 (14%)
Probability of buying a product with Pp_OE_2_P odour						
1. Would never buy	50 (50%)	25 (25%)	18 (18%)	39 (39%)	34 (34%)	47 (47%)
2. Unlikely	34 (34%)	35 (35%)	38 (38%)	37 (37%)	35 (35%)	35 (35%)
3. Likely	12 (12%)	28 (28%)	29 (29%)	19 (19%)	22 (22%)	13 (13%)
4. Quite likely	3 (3%)	8 (8%)	11 (11%)	5 (5%)	6 (6%)	2 (2%)
5. Would buy	1 (1%)	4 (4%)	4 (4%)	0 (0%)	3 (3%)	3 (3%)
Probability of buying a product with Pp_OE_1_G odour						
1. Would never buy	54 (54%)	32 (32%)	27 (27%)	42 (42%)	38 (38%)	54 (54%)
2. Unlikely	32 (32%)	40 (40%)	33 (33%)	40 (40%)	34 (34%)	39 (39%)
3. Likely	10 (10%)	19 (19%)	28 (28%)	13 (13%)	18 (18%)	6 (6%)
4. Quite likely	1 (1%)	3 (3%)	6 (6%)	2 (2%)	5 (5%)	1 (1%)
5. Would buy	3 (3%)	6 (6%)	6 (6%)	3 (3%)	5 (5%)	0 (0%)

**Table 9:** Participants responses to other applications of the emulsions’ odours.

Section 2. Applicability of Emulsions’ Odour. Other Applicability of Emulsions’ Odour	N (%)
Aromatherapy and bath bombs	1 (1%)
Soaps	2 (2%)
Cleaning products	10 (10%)
Massage oils	1 (1%)
Repellents	2 (2%)
Nasal spray	1 (1%)
Incense and cleaning products	1 (1%)
Shower gel	1 (1%)
Ointment medications (analgesics)	3 (3%)
Wood Furniture Cleaning Products	1 (1%)
Hand and face cream	1 (1%)
Disinfectant	1 (1%)
Body and hand cream	1 (1%)
Deodorant	2 (2%)
Candles and Soaps	1 (1%)
Shaving cream	1 (1%)
Nasal decongestant	2 (2%)
Car air freshener and cleaning products	1 (1%)
None	9 (9%)

**Table 10:** Analyzed essential oils (EOs) and hydrolates (Hds) and their codes.

Plant Species	EOs Code *	Hds Code
*Eucalyptus globulus*	Eg_OE_1_G	Eg_Hd_1_G
	Eg_OE_2_B	-
	Eg_OE_3_O	Eg_Hd_2_O
	Eg_OE_4_E	Eg_Hd_3_E
	Eg_OE_5_P	Eg_Hd_4_P
	Eg_OE_6_S	-
*Pinus pinaster*	Pp_OE_1_G	Pp_Hd_1_G
	Pp_OE_2_P	Pp_Hd_2_P
	Pp_OE_3_S	-
*Pinus pinea*	Ppi_OE_1_B	-
*Cryptomeria japonica*	Cj_OE_1_M	Cj_Hd_1_M

* To ensure data protection each producer was assigned with an arbitrary code letter.

**Table 11:** Qualitative and quantitative (%, *w*/*w*) composition of the emulsions prepared with *Eucalyptus globulus*, *Pinus pinaster*, *Pinus pinea* and *Cryptomeria japonica* essential oils.

INCI *	Trade Name	Function	(%, *w*/*w*)
Phase A			
Ceteareth-11	Eumulgin B1^®^	Non-ionic O/W emulsifier	1.5
Ceteareth-20	Eumulgin $B2^{®}$	Non-ionic O/W emulsifier	1.5
Cetyl alcohol	Cetyl alcohol	Thickener	2.0
*Paraffinum liquidum*	Mineral oil	Emollient	2.5
Decyl oleate	Tegosoft DO^®^		4.5
Phase B			
Glycerin	Glycerin	Humectant	5.0
Aqua	Purified water	Solvent	82.0
Phase C			
Parfum	Essential Oil	Fragrance	0.5

* Ingredients’ names according to the International Nomenclature of Cosmetics Ingredients (INCI).

## Data Availability

Not applicable.

## Supplementary Materials

The following supporting information can be downloaded at: https://www.mdpi.com/article/10.3390/molecules27113572/s1, Table S1. Percentage composition of *Eucalyptus globulus* essential oils. For samples codes *vide* Table 10 in Materials and Methods section. Table S2. Percentage composition of *Pinus pinaster* essential oils. For samples codes *vide* Table 10 in Materials and Methods section. Table S3. Percentage composition of *Pinus pinea* essential oil. For samples codes *vide* Table 10 in Materials and Methods section. Table S4. Percentage composition of *Cryptomeria japonica* essential oil. For samples codes *vide* Table 10 in Materials and Methods section. Table S5. Percentage composition of *Eucalyptus globulus* hydrolates volatiles. For samples codes *vide* Table 10 in Materials and Methods section. Table S6. Percentage composition of *Pinus pinaster* hydrolates volatiles. For samples codes *vide* Table 10 in Materials and Methods section. Table S7. Percentage composition of *Cryptomeria japonica* hydrolates volatiles. For samples codes *vide* Table 10 in Materials and Methods section. Sensory Questionnaire (English Version).

## Sample Availability

Samples of EOs and Hds are available from the authors.
