# Supplementary material for: Chemical Characterization and Bioactivity of Commercial Essential Oils and Hydrolates Obtained from Portuguese Forest Logging and Thinning
Source: Molecules. 2022 Jun 2;27(11):3572. doi: 10.3390/molecules27113572 (PMC9181877; doi:10.3390/molecules27113572)
Supplement: Supplementary file 1 [file molecules-27-03572-s001.zip › molecules-1747665-supplementary.pdf]

Article

Chemical Characterization and Bioactivity of Commercial Essential Oils and Hydrolates Obtained from Portuguese Forest Logging and Thinning

Supplementary Material

Essential oils composition

Table S1. Percentage composition of *Eucalyptus globulus* essential oils. For samples codes *vide* Table 10 in Materials and Methods section.

Components	RI	Eg_OE_1_G	Eg_OE_2_B	Eg_OE_3_O	Eg_OE_4_E	Eg_OE_5_P	Eg_OE_6_S
Isovaleraldehyde	637	0.1	0.4	t	t	t	0.1
Isoamyl alcohol	722	t	t	t	t	t	t
Isovaleric acid	847	t	t	t	t	t	
Isoamyl acetate	882	t	t	t	t	t	t
α-Thujene	924	t	t	t	t	t	t
α-Pinene	930	13.2	13.3	11.0	21.8	14.7	13.8
α-Fenchene	938	t	0.1	t	0.1	0.1	0.1
Camphene	938	t	0.1	t	0.1	0.1	0.1
Thuja-2,4(10)-diene*	940	t	t	t	t	t	t
β-Pinene	963	0.4	0.3	0.4	0.7	0.4	0.3
Dehydro 1,8-cineole	973	t	t	t	t	t	t
β-Myrcene	975	0.2	t	1.0	0.7	0.1	t
α-Phellandrene	995	0.3	0.1	0.2	0.2	0.3	0.3
α-Terpinene	1002	t		0.1	0.1	t	t
p-Cymene	1003	1.2	0.8	0.2	0.6	1.6	1.7
1,8-Cineole	1005	65.2	63.2	59.5	53.9	58.2	49.4
Limonene	1009	8.2	17.2	13.7	16.6	12.5	18.0
cis-β-Ocimene	1017	0.3	t	0.2	0.5	0.1	t
trans-β-Ocimene	1027	t	0.1	t	0.1	t	
γ-Terpinene	1035	0.5	t	0.7	0.9	0.2	0.2
2,5-Dimethyl styrene	1059	t	t	t	t	0.2	0.2
Terpinolene	1064	t	t	0.2	0.1	0.1	0.1
Nonanal	1073	t	t	t	t	t	t
Linalool	1074	t		t	t	t	t
Isopentyl isovalerate	1080	t	t	t	t	0.1	0.1
endo-Fenchol	1085	t	t	t	t	0.1	0.1
α-Campholenal	1092	0.2	0.1	t	t	0.1	0.1
Cosmene*	1102	t	t			t	t
trans-Pinocarveol	1106	1.6	1.9	t	0.3	3.0	3.1
Pinocarvone	1121	1.3	1.2	t	0.1	1.3	1.3
δ-Terpineol	1134	0.1	t	0.1	0.1	0.1	0.2
Borneol	1138	0.1	t	0.1	0.1	0.1	0.2
Terpinen-4-ol	1148	0.3	t	0.5	0.3	0.2	0.2
α-Terpineol	1159	0.6	0.2	2.0	1.3	0.3	0.7
Myrtenol	1168	t	t	t	t	0.1	0.2
cis-Piperitol*	1182	t	t	t	t	t	t

Components	RI	Eg_OE_1_G	Eg_OE_2_B	Eg_OE_3_O	Eg_OE_4_E	Eg_OE_5_P	Eg_OE_6_S
cis-carveol	1202	0.1	t	t	t	0.1	0.1
Carvone	1210	t	t		t	t	0.1
Geraniol	1236	0.2		0.5	t	t	t
α-Terpenyl acetate	1334	2.2	t	5.4	0.2	0.8	0.9
Geranyl acetate	1370	t		0.2	t	t	t
β-Caryophyllene	1414	0.6	t	t	t	0.2	0.2
Aromadendrene	1428	1.2	0.4	0.4	0.4	3.1	4.3
α-Humulene	1447	0.3	t	t	t	t	0.1
allo-Aromadendrene	1456	0.3	t	0.2	0.1	0.6	0.8
Phenethyl isovalerate	1468	t	t	0.1	t	t	t
Germacrene-D	1474	t		t		t	0.1
Viridiflorene	1487	t	t	0.2	t	0.2	0.3
Globulol	1566	0.3	0.2	0.7	0.2	0.5	1.0
**% Identification**		99.0	99.6	97.6	99.5	99.5	99.4
**Grouped components**:							
Monoterpene hydrocarbons		24.3	32.0	27.7	42.5	30.2	34.6
Oxygen-containing monoterpenes		71.9	66.6	68.3	56.3	64.4	56.6
Sesquiterpene hydrocarbons		2.4	0.4	0.8	0.5	4.1	5.8
Oxygen-containing sesquiterpenes		0.3	0.2	0.7	0.2	0.5	1.0
Others.		0.1	0.4	0.1	t	0.3	0.4

RI: In-lab calculated retention index relative to C_9-C_{16} *n*-alkanes on the DB-1 column. t: traces (< 0.05%). * Identification based on mass spectra only.

Table S2. Percentage composition of *Pinus pinaster* essential oils. For samples codes *vide* Table 10 in Materials and Methods section.

Components	RI	Pp_OE_1_G	Pp_OE_2_P	Pp_OE_3_S
Hexenal	866			t
cis-3-Hexen-1-ol	868		t	t
Tricyclene	921	t	t	t
α-Thujene	924	t	t	t
α-Pinene	930	28.0	44.6	36.5
α-Fenchene	938	0.2	0.4	0.4
Camphene	938	0.2	0.4	0.4
Thuja-2,4(10)-diene*	940	0.1	0.1	0.3
Sabinene	958	t	t	t
β-Pinene	963	28.5	23.0	18.8
2-Pentyl furan	973	t	t	t
β-Myrcene	975	11.0	5.0	5.9
α-Phellandrene	995	0.1	t	0.2
δ-3-Carene	1000	6.6	2.1	1.8
α-Terpinene	1002	0.1	t	0.1
p-Cymene	1003	0.1	0.2	0.2
β-Phellandrene	1005	1.1	0.8	1.2
1,8-Cineole	1005		0.8	0.6
Limonene	1009	4.5	3.9	3.3
cis-β-Ocimene	1017	t	t	t
trans-β-Ocimene	1027	0.6	0.2	0.5
γ-Terpinene	1035	0.1	t	t
2,5-Dimethyl styrene	1059	t	0.1	0.1
Terpinolene	1064	1.1	0.3	0.7

Components	RI	Pp_OE_1_G	Pp_OE_2_P	Pp_OE_3_S
Nonanal	1073	t	0.3	0.1
Linalool	1074	t	0.1	0.1
Isopenthyl isovalerate	1080	t	t	t
endo-Fenchol	1085	0.1	0.1	0.1
trans-Pinocarveol	1106	t	0.4	0.1
cis-Verbenol	1113	t	0.1	t
trans-Pinocamphone	1121	t	0.1	0.1
Borneol	1138	t	0.1	0.2
Terpinen-4-ol	1148	t	t	0.1
p-Cymen-8-ol	1148		t	t
Myrtenal	1153	t	0.1	0.1
α-Terpineol	1159	0.3	0.5	0.6
Myrtenol	1168		0.2	0.1
Methyl thymol	1210	0.1	t	0.1
Hexyl isovalerate	1225	t	0.1	0.1
Linalyl acetate	1245	0.1	0.1	0.1
Bornyl acetate	1265	0.1	0.1	0.1
Thymol	1275	t		t
trans-Pinocarvyl acetate	1278	t	t	t
Tridecane	1300	t	t	t
α-Cubebene	1345	0.2	0.4	0.4
Geranyl acetate	1370	0.1	0.1	0.1
α-Ylangene	1371	0.1	0.1	0.1
α-Copaene	1375	0.3	0.4	0.6
β-Bourbonene	1379	t	0.2	t
Longifolene	1399	0.4	0.9	1.4
β-Caryophyllene	1414	4.5	5.0	8.7
β-Copaene	1426	0.1	0.2	0.3
Aromandendrene	1428		0.1	
α-Humulene	1447	0.6	0.7	1.2
Phenethyl 2-methyl butyrate	1467	0.1	0.1	0.1
Phenethyl isovalerate	1468	0.3	0.1	0.4
γ-Muurolene	1469	0.7	1.0	1.4
Germacrene-D	1474	6.3	1.7	5.6
α-Muurolene	1494	0.2	0.3	0.5
γ-Cadinene	1500	0.4	0.4	0.4
trans-Calamenene	1505	t	0.2	0.1
δ-Cadinene	1505	1.1	1.0	2.1
α-Cadinene	1529	t	t	0.1
trans-α-Bisabolene	1536	0.1	0.1	0.1
β-Caryophyllene oxide	1561	0.1	0.7	0.2
Abietatriene	2045	t	0.2	t
Abietadiene	2060	0.4	0.2	0.5
Abieta-8(14),13(15)-diene*	2116	0.1	t	0.1
**% Identification**		99.1	98.4	97.7
**Grouped components**				
Monoterpene hydrocarbons		82.3	81.0	70.3
Oxygen-containing monoterpenes		0.8	2.9	2.8
Sesquiterpene hydrocarbons		15.0	12.7	23.0
Oxygen-containing sesquiterpenes		0.1	0.7	0.2
Diterpene hydrocarbons		0.5	0.4	0.6
Others.		0.4	0.7	0.8

RI: In-lab calculated retention index relative to C_9-C_{22} *n*-alkanes on the DB-1 column. t: traces (< 0.05%). * Identification based on mass spectra only.

Table S3. Percentage composition of *Pinus pinea* essential oil. For samples codes *vide* Table 10 in Materials and Methods section.

Components	RI	Ppi_OE_1_B
2-*trans*-Hexenal	866	t
cis-3-Hexen-1-ol	868	0.2
n-Hexanol	881	t
Tricyclene	921	t
α-Thujene	924	t
α-Pinene	930	7.6
Camphene	938	0.1
Thuja-2,4(10)-diene*	940	t
Sabinene	958	t
β-Pinene	963	1.2
β-Myrcene	975	2.1
α-Phellandrene	995	0.1
p-Cymene	1003	0.3
1,8-Cineole	1005	3.8
β-Phellandrene	1005	3.8
Limonene	1009	72.8
cis-β-Ocimene	1017	0.1
trans-β-Ocimene	1027	t
γ-Terpinene	1035	t
2,5-Dimethyl styrene	1059	t
Terpinolene	1064	0.1
n-Nonanal	1073	t
Linalool	1074	t
endo-Fenchol	1085	t
cis-Limonene oxide	1095	0.2
trans-Limonene oxide	1112	0.1
Cryptone*	1143	0.3
Terpinen-4-ol	1148	0.1
α-Terpineol	1159	0.3
trans-Carveol	1189	0.2
cis-Carveol	1202	0.1
Carvone	1210	0.2
Methyl thymol	1210	0.6
Bornyl acetate	1265	0.1
α-Terpenyl acetate	1334	0.6
α-Longipinene	1338	0.5
Longifolene	1399	1.0
β-Caryophyllene	1414	1.0
β-Copaene	1426	t
Aromadendrene	1428	0.6
α-Humulene	1447	0.2
trans-β-Farnesene	1455	0.1
allo-Aromadendrene	1456	0.1
Phenethyl 2-methyl butyrate	1467	0.1
γ-Muurolene	1469	t

Components	RI	Ppi_OE_1_B
Germacrene-D	1474	t
Viridiflorene	1487	0.1
β-Caryophyllene oxide	1561	0.3
Globulol	1566	0.2
Viridiflorol	1569	t
**% Identification**		99.2
**Grouped components**		
Monoterpene hydrocarbons		88.2
Oxygen-containing monoterpenes		6.6
Sesquiterpene hydrocarbons		3.6
Oxygen-containing sesquiterpenes		0.5
Others.		0.3

RI: In-lab calculated retention index relative to C_8-C_{16} *n*-alkanes on the DB-1 column. t: traces (< 0.05%). * Identification based on mass spectra only.

Table S4. Percentage composition of *Cryptomeria japonica* essential oil. For samples codes *vide* Table 10 in Materials and Methods section.

Components	RI	Cj_OE_1_M
Tricyclene	921	0.3
α-Thujene	924	1.3
α-Pinene	930	26.1
α-Fenchene	938	0.6
Camphene	938	1.8
Sabinene	958	18.1
β-Pinene	963	2.0
β-Myrcene	975	4.4
α-Phellandrene	995	0.1
δ-3-Carene	1000	1.2
α-Terpinene	1002	1.6
p-Cymene	1003	0.4
β-Phellandrene	1005	0.7
Limonene	1009	3.8
cis-β-Ocymene	1017	t
trans-β-Ocymene	1027	t
γ-Terpinene	1035	2.6
trans-Sabinene hydrate	1037	0.2
2,5-Dimethyl styrene	1059	t
Terpinolene	1064	1.0
cis-Sabinene hydrate	1066	0.1
Linalool	1074	0.2
trans-Thujone	1081	t
1-Octen-3-yl-acetate	1086	t
α-Campholenal	1092	0.1
trans-*p*-2-Menthen-1-ol	1099	0.1
Camphor	1102	t
cis-*p*-2-Menthen-1-ol	1114	0.1
trans-Pinocamphone	1121	t
Borneol	1138	t
cis-Pinocamphone	1134	t
Terpinen-4-ol	1148	2.3

Components	RI	Cj_OE_1_M
α-Terpineol	1159	0.1
cis-Piperitol*	1182	t
trans-Piperitol	1189	t
α-Fenchyl acetate	1200	t
Piperitone	1211	t
Geraniol	1236	t
Linalyl acetate	1245	0.2
trans-Anethole	1254	t
Bornyl acetate	1265	1.8
cis-Verbenyl acetate	1266	0.1
α-Terpenyl acetate	1334	0.2
α-Cubebene	1345	t
Geranyl acetate	1370	t
α-Copaene	1375	t
β-Bourbonene	1379	t
β-Elemene	1388	0.1
β-Caryophyllene	1414	t
β-Copaene	1426	0.1
α-Humulene	1447	t
trans-β-Farnesene	1455	t
γ-Muurolene	1469	0.1
Germacrene-D	1474	0.3
α-Muurolene	1494	0.1
β-Bisabolene	1500	0.2
γ-Cadinene	1500	0.2
δ-Cadinene	1505	0.5
α-Cadinene	1529	t
Elemol	1530	4.4
trans-Nerolidol	1549	t
Germacrene-D-4-ol*	1557	0.2
Cedrol	1574	t
Anydrooplopanone	1576	0.1
10-epi-γ-Eudesmol	1593	0.1
γ-Eudesmol	1609	0.6
trans-Muurolol	1616	0.1
α-Muurolol	1618	0.1
β-Eudesmol	1620	0.9
α-Eudesmol	1634	1.1
Cryptomerione*	1686	t
Oplopanoyl acetate*	1808	t
Rimuene	1814	t
Isopimara-9(11),15-diene	1821	0.5
Isokaurene*	1977	0.5
Sandaracopimara-8(14),15-diene	1956	0.7
Phyllocladene	2006	13.8
Kaurene	2044	0.5
Nezukol*	2112	0.7
**% Identification**		97.4
**Grouped components**		
Monoterpene hydrocarbons		66.0
Oxygen-containing monoterpenes		5.5
Sesquiterpene hydrocarbons		1.6

Components	RI	Cj_OE_1_M
Oxygen-containing sesquiterpenes		7.6
Diterpene hydrocarbons		16.0
Oxygen-containing diterpenes		0.7
Phenylpropanoids		t
Others.		t

RI: In-lab calculated retention index relative to C_9-C_{22} *n*-alkanes on the DB-1 column. t: traces (< 0.05%). * Identification based on mass spectra only.

Hydrolates volatiles composition

Table S5. Percentage composition of *Eucalyptus globulus* hydrolates volatiles. For samples codes *vide* Table 10 in Materials and Methods section.

Components	RI	Eg_Hd_1_G	Eg_Hd_2_O	Eg_Hd_3_E	Eg_Hd_4_P
2-*trans*-Hexenal	866	t	t	t	t
Isovaleric acid	867	t	t	t	t
cis-3-Hexen-1-ol	868	t	t	t	t
2-Methyl butyric acid	871	t		t	t
cis-2-Hexen-1-ol	882	t	t	t	t
n-Hexanol	883	t	t	t	t
2-Acetyl furan*	897				t
Terbutyl isovalerate	924	t		t	t
Benzaldehyde	927		t		
α-Pinene	930	t		t	t
Hexanoic acid	968	t	t	t	t
Benzyl alcohol	1002		t	t	t
Benzene acetaldehyde	1004		t	t	t
2-Ethyl-1-hexanol	1004				2.3
1,8-Cineole	1005	80.2	55.5	53.5	4.5
Limonene	1009	7.3	14.1	6.7	1.5
Acetophenone	1017	t	t	t	
cis-Linalool oxide (furanoid)	1045	t	0.1	t	t
trans-Linalool oxide (furanoid)	1059	t	0.1	t	t
Phenyl ethyl alcohol	1067	t	0.1		t
Linalool	1074	t	0.5	t	t
endo-Fenchol	1085	t	t	t	t
α-Campholenal	1092	t	t	t	0.4
Nopinone	1093	t	t		t
trans-*p*-2-Menthen-1-ol	1099	t	t	t	t
Cosmene*	1102	t		t	t
trans-Pinocarveol	1106	4.9	0.4	8.0	36.6
cis-Verbenol	1114	t	0.1	t	
cis-*p*-2-Menthen-1-ol	1114	t	t	t	4.6
Pinocarvone	1121	1.6	0.1	t	0.3
Benzyl acetate	1123		t		
δ-Terpineol	1134	t	1.2		
Borneol	1138	t	1.2		3.7
p-Cymen-8-ol	1148	0.9	t	t	2.4
Terpinen-4-ol	1148	t	3.0	2.2	
Myrtenal	1153		t	t	5.5
α-Terpineol	1159	2.7	17.2	24.7	5.3
Verbenone	1164	t	t	t	

Components	RI	Eg_Hd_1_G	Eg_Hd_2_O	Eg_Hd_3_E	Eg_Hd_4_P
Myrtenol	1168		t	t	12.0
trans-Carveol	1189	t	0.1	t	2.4
cis-Carveol	1202	1.1	0.1	2.8	8.6
2-Hydroxy-3-pinanone	1206				t
Carvone	1210	t		t	t
Neral	1210	t	t	t	
Citronellol	1211	t	0.5		
cis-Piperitone epoxide	1211	t	0.4	t	0.9
2-Phenyl ethyl acetate	1228	t	t	t	
Geraniol	1236	t	2.6	t	t
m-Acetanisole	1237	t	0.5	t	
Geranial	1240	t	0.2	t	t
Linalyl acetate	1254		t	t	t
Thymol	1275	t	t	t	1.5
2-Methoxy-4-vinylphenol	1285	t	t	t	t
Carvacrol	1286	t	t	t	t
p-Acetanisole *	1311	t	t	t	
Exo-2-hydroxy cineole acetate *	1323	t	0.8		2.4
α-Terpenyl acetate	1334		t	t	
Geranyl acetate	1370	t	t	t	
Perilla alcohol isopentyl ether	1389		t	t	t
Spathulenol	1551	t	t	t	
Viridiflorol	1569	t	t	t	
Globulol	1566	t	t	t	t
Ledol	1580		0.2	t	
γ-Eudesmol	1609	t	0.1	t	
β-Eudesmol	1620	t	0.2		
α-Eudesmol	1634	t	0.1		
**% Identification**		98.8	99.2	97.8	94.7
**Grouped components:**					
Monoterpene hydrocarbons		7.3	14.3	6.7	1.5
Oxygen-containing monoterpenes		91.5	83.8	91.1	91.0
Oxygen-containing sesquiterpenes		0.0	0.5	t	t
Others.		t	0.6	t	2.3

RI: In-lab calculated retention index relative to C_8-C_{17} *n*-alkanes on the DB-1 column. t: traces (< 0.05%). * Identification based on mass spectra only.

Table S6. Percentage composition of *Pinus pinaster* hydrolates volatiles. For samples codes *vide* Table 10 in Materials and Methods section.

Components	RI	Pp_Hd_1_G	Pp_Hd_2_P
2-*trans*-Hexenal	866	t	t
cis-3-Hexen-1-ol	868	t	t
cis-2-Hexen-1-ol	882	t	t
n-Hexanol	883	t	t
Benzaldehyde	927	t	t
α-Pinene	930	t	t
β-Pinene	963	t	t
Benzyl alcohol	1002	t	t
Benzene acetaldehyde	1004	t	t
2-Ethyl-1-hexanol	1004		t

Components	RI	Pp_Hd_1_G	Pp_Hd_2_P
1,8-Cineole	1005	5.0	t
Limonene	1009	4.3	
cis-Linalool oxide (furanoid)	1045		t
trans-Linalool oxide (furanoid)	1059		t
Phenyl ethyl alcohol	1067		t
Linalool	1074	t	t
endo-Fenchol	1085	t	t
trans-*p*-2-Menthen-1-ol	1099	t	t
Camphor	1102	t	t
trans-Pinocarveol	1106	t	t
cis-Verbenol	1114	t	t
cis-*p*-2-Menthen-1-ol	1114	t	14.0
neo-Isopulegol	1116		14.0
trans-Pinocamphone	1121	t	t
Pinocarvone	1121	t	t
Isoborneol	1132	t	t
cis-Linalool oxide (pyranoid)	1132		t
Borneol	1138	t	t
Terpinen-4-ol	1148	7.5	
p-Cymen-8-ol	1148	7.5	t
Myrtenal	1153	t	
α-Terpineol	1159	43.8	38.1
Verbenone	1164	17.9	28.7
trans-Carveol	1189	t	t
Geraniol	1236	t	t
Perilla alcohol	1274	6.6	t
Thymol	1275	6.6	t
Carvacrol	1286	t	t
Geranyl acetate	1370	t	t
Methyl eugenol	1377	t	t
Phenethyl 2-methybutyrate	1467	t	t
Phenethyl isovalerate	1468		t
Globulol	1566	t	t
epi-α-Muurolol	1616	t	t
α-Cadinol	1616	t	t
**% Identification**		99.1	94.7
**Grouped components**			
Monoterpene hydrocarbons		4.3	t
Oxygen-containing monoterpenes		94.8	94.7
Oxygen-containing sesquiterpenes		0.0	t
Phenylpropanoids		t	t
Others.		t	t

RI: In-lab calculated retention index relative to C_8-C_{17} *n*-alkanes on the DB-1 column. t: traces (< 0.05%). * Identification based on mass spectra only.

Table S7. Percentage composition of *Cryptomeria japonica* hydrolates volatiles. For samples codes *vide* Table 10 in Materials and Methods section.

Components	RI	Cj_Hd_1_M
cis-3-Hexen-1-ol	868	t
n-Hexanol	883	t

Components	RI	Cj_Hd_1_M
n-Nonane	900	t
α-Pinene	930	t
n-Heptanol	952	t
1-Octen-3-ol	961	t
β-Pinene	963	t
β-Myrcene	975	t
α-Phellandrene	995	t
Benzene acetaldehyde	1002	t
α-Terpinene	1002	t
p-Cymene	1003	t
1,8-Cineole	1005	6.3
Limonene	1009	2.3
Acetophenone	1017	t
γ-Terpinene	1035	t
cis-Linalool oxide (furanoid)	1045	t
Fenchone	1050	t
trans-Linalool oxide (furanoid)	1059	t
Linalool	1074	2.6
trans-p-2-Menthen-1-ol	1099	2.8
Camphor	1102	0.4
cis-p-2-Menthen-1-ol	1114	2.5
Pinocarvone	1121	0.7
cis-Pinocamphone	1134	0.3
Borneol ethyl ether	1138	0.7
Terpinen-4-ol	1148	56.2
α-Terpineol	1159	4.6
Citronellol	1211	0.2
Linalyl acetate	1245	0.5
Bornyl acetate	1265	0.8
trans-Pinocarvyl acetate	1278	t
Carvacrol	1286	0.1
trans-Carvyl acetate	1305	t
α-Terpenyl acetate	1334	t
Vanillin	1358	t
Elemol	1530	4.2
Anydrooplopanone	1576	t
γ-Eudesmol	1609	1.6
β-Eudesmol	1620	1.6
Valerianol	1623	0.2
α-Eudesmol	1634	2.1
Oplopanoyl acetate*	1808	t
Phyllocladene	2006	4.8
**% Identification**		95.4
**Grouped components**		
Monoterpene hydrocarbons		2.3
Oxygen-containing monoterpenes		78.7
Oxygen-containing sesquiterpenes		9.6
Diterpene hydrocarbons		4.8
Others.		t

RI: In-lab calculated retention index relative to C_8-C_{21} *n*-alkanes on the DB-1 column. t: traces (< 0.05%). * Identification based on mass spectra only.

Sensorial evaluation

Sensory Questionnaire (English Version)

The questionnaire consists of short and objective questions that must be answered based on the samples provided. The data collected is anonymous and is used only for the mentioned purpose.

1. The aroma of many plant species has different volatile chemical compounds with the ability to stimulate our olfactory receptors. The characteristic aromas of plants are related to the quantity and diversity of these compounds in plant species.

2. Essential oils (EOs) are mixtures of volatile compounds isolated from different parts of plants and there is an increasing interest in their use for different purposes, such as in the cosmetics, perfumery, and aromatherapy industries.

Age:
18-30 years old
31-40 years old
41-50 years old
51-60 years old
More than 60 years old

Gender:
Female
Male

Education level:
Primary education
Secondary education
Higher education

Region:
Country
City

Section 1. Emulsion's odour

1. How do you evaluate the odour of the "pink" emulsion?
- Without odour
- Slightly perceptible
- Perceptible
- Very perceptible
- Intense odour

2. In case you identified any odour, how would you classify it?
- Very unpleasant
- Unpleasant
- Pleasant and fresh odour
- Pleasant and hot odour

3. How do you evaluate the odour of the "green" emulsion?
- Without odour
- Slightly perceptible
- Perceptible
- Very perceptible

- Intense odour

4. In case you have identified any odour, how would classify it?
- Very unpleasant
- Unpleasant
- Pleasant and fresh odour
- Pleasant and hot odour

5. How do you evaluate the odour of the "orange" emulsion?
- Without odour
- Slightly perceptible
- Perceptible
- Very perceptible
- Intense odour

6. In case you have identified any odour, how would you classify it?
- Very unpleasant
- Unpleasant
- Pleasant and fresh odour
- Pleasant and hot odour

7. How do you evaluate the odour of the "purple" emulsion?
- Without odour
- Slightly perceptible
- Perceptible
- Very perceptible
- Intense odour

8. In case you identified any odour, how would you classify it?
- Very unpleasant
- Unpleasant
- Pleasant and fresh odour
- Pleasant and hot odour

9. How do you evaluate the odour of the "blue" emulsion?
- Without odour
- Slightly perceptible
- Perceptible
- Very perceptible
- Intense odour

10. In case you have identified any odour, how would you classify it?
- Very unpleasant
- Unpleasant
- Pleasant and fresh odour
- Pleasant and hot odour

11. In your opinion the odours of the different emulsions belong to the same plant species?
- Yes
- No
- Maybe

12. Select which emulsion(s) cause you a feeling of physical and/or mental well-being.

- Pink
- Green
- Orange
- Purple
- Blue
- None

13. Refers what feeling of well-being the emulsions caused you.
- Relaxing
- Decongestant
- Stimulating
- Refreshing
- None

14. Order the samples, according to your preference, on a scale of 1-5 (1-Hateful odour; 2-Unpleasant odour; 3-Pleasant odour; 4-Very pleasant odour and 5-Favourite odour).

	1	2	3	4	5
Pink					
Blue					
Purple					
Green					
Orange					

Section 2. Emulsion's Applicability's

1. Rate each of the products below, on a scale of 1-5: 1. Would never buy, 2. Unlikely, 3. Likely, 4. Quite likely and 5. Would buy, considering the probability of buying one with the "pink" emulsion odour

	1	2	3	4	5
Perfume					
Air freshener					
Massage cream					
Toothpaste					
Shampoo					
Candy					

2. Rate each of the products below, on a scale of 1-5: 1. Would never buy, 2. Unlikely, 3. Likely, 4. Quite likely and 5. Would buy, considering the probability of buying one with the "green" emulsion odour

	1	2	3	4	5
Perfume					
Air freshener					
Massage cream					
Toothpaste					
Shampoo					
Candy					

3. Rate each of the products below, on a scale of 1-5: 1. Would never buy, 2. Unlikely, 3. Likely, 4. Quite likely and 5. Would buy, considering the probability of buying one with the "orange" emulsion odour

	1	2	3	4	5
Perfume					

Air freshener					
Massage cream					
Toothpaste					
Shampoo					
Candy					

4. Rate each of the products below, on a scale of 1-5: 1. Would never buy, 2. Unlikely, 3. Likely, 4. Quite likely and 5. Would buy, considering the probability of buying one with the "purple" emulsion odour

	1	2	3	4	5
Perfume					
Air freshener					
Massage cream					
Toothpaste					
Shampoo					
Candy					

5. Rate each of the products below, on a scale of 1-5: 1. Would never buy, 2. Unlikely, 3. Likely, 4. Quite likely and 5. Would buy, considering the probability of buying one with the "blue" emulsion odour

	1	2	3	4	5
Perfume					
Air freshener					
Massage cream					
Toothpaste					
Shampoo					
Candy					

6. Do you consider that the odours in the samples have other applicability? If yes, refer which/which samples and the respective applicability

Answer: ____
